# Geographic disparities in Saskatchewan prostate cancer incidence and its association with physician density: analysis using Bayesian models

**DOI:** 10.1186/s12885-021-08646-2

**Published:** 2021-08-23

**Authors:** Mustafa Andkhoie, Michael Szafron

**Affiliations:** University of Saskatchewan, 104 Clinic Place, Saskatoon, SK S7N 2Z4 Canada

**Keywords:** Prostate cancer, Prostatic neoplasms, Geography, Epidemiology, Spatial analysis, Physician supply, Healthcare access

## Abstract

**Background:**

Saskatchewan has one of the highest incidence of prostate cancer (PCa) in Canada. This study assesses if geographic factors in Saskatchewan, including location of where patients live and physician density are affecting the PCa incidence. First, the objective of this study is to estimate the PCa standardized incidence ratio (SIRs) in Saskatchewan stratified by PCa risk-level. Second, this study identifies clusters of higher than and lower than expected PCa SIRs in Saskatchewan. Lastly, this study identifies the association (if any) between family physician density and estimated PCa SIRs in Saskatchewan.

**Methods:**

First, using Global Moran’s I, Local Moran’s I, and the Kuldorff’s Spatial Scan Statistic, the study identifies clusters of PCa stratified by risk-levels. Then this study estimates the SIRs of PCa and its association with family physician density in Saskatchewan using the Besag, York, and Mollie (BYM) Bayesian method.

**Results:**

Higher than expected clusters of crude estimated SIR for metastatic PCa were identified in north-east Saskatchewan and lower than expected clusters were identified in south-east Saskatchewan. Areas in north-west Saskatchewan have lower than expected crude estimated SIRs for both intermediate-risk and low-risk PCa. Family physician density was negatively associated with SIRs of metastatic PCa (IRR: 0.935 [CrI: 0.880 to 0.998]) and SIRs of high-risk PCa (IRR: 0.927 [CrI: 0.880 to 0.975]).

**Conclusions:**

This study identifies the geographical disparities in risk-stratified PCa incidence in Saskatchewan. The study identifies areas with a lower family physician density have a higher-than-expected incidences of metastatic and high-risk PCa. Hence policies to increase the number of physicians should ensure an equitable geographic distribution of primary care physicians to support early detection of diseases, including PCa.

## Background

Prostate cancer (PCa) accounts for about 20% of all new cancer cases among men in Canada [1]. Within Canada, the incidence rate of PCa varies between provinces. In 2019, Saskatchewan had the third highest projected age-standardized PCa incidence rate (117.8 cases per 100,000 in 2019) when compared to other Canadian provinces [1]. In addition, the Saskatchewan age-standardized PCa incidence rates have remained higher than the national Canadian rates for the majority of the past 10 years [1, 2]. Previous studies have shown geographic factors influence PCa outcomes in Saskatchewan [3, 4], hence this study explores the influence of geographic patterns on PCa incidence rates in Saskatchewan.

Saskatchewan, in terms of geography, has the second lowest population density in Canada (after Newfoundland), with a majority of province sparsely populated and nearly 40% of the Saskatchewan population living in rural areas [5]. Because cancer patient outcomes are worse for rural dwellers compared to urban dwellers [6–10] and Saskatchewan has a relatively large rural population [5], it is possible that the rates for different PCa risk-levels are associated with the geographic distribution of Saskatchewan residents.

The low population density of Saskatchewan results in the geographic factors of remoteness and commute time being healthcare access barriers for Saskatchewan residents [3, 4, 11, 12]. The low population density also impacts the distribution of physicians in the province. Saskatchewan has one of the lowest per capita physician supplies (also known as physician density) compared to the other provinces in Canada (190.3 per 100,000 in 2014 and 204.5 per 100,000 in 2018) [13]. Because one mechanism for improving health outcomes, including reductions in PCa-specific mortalities, is increasing physician supply [14–20], understanding the association (if any) between physician density and PCa risk-level incidence is crucial to improving PCA outcomes in Saskatchewan.

While the factors leading to such high Saskatchewan PCa incidence rates are unknown, we hypothesize that the unique geography of Saskatchewan may be contributing to the high incidence of PCa in Saskatchewan. In this study we explore the geographic distribution of PCa cases in Saskatchewan. In addition, since the incidence for advanced cancers is known to decrease with the increase in availability of physicians [18, 21, 22], we identify the association (if any) that exists between the family physician density and PCa standardized incidence ratios (SIRs) in Saskatchewan.

The first study objective is to estimate the PCa SIRs in Saskatchewan stratified by PCa risk-levels. The second objective is to identify clusters of higher than and lower than expected PCa SIR stratified by PCa risk-levels in Saskatchewan. The final objective is to identify any associations between family physician density and estimated PCa SIRs in Saskatchewan.

## Methods

### Data and study area

**Fig. 1:**
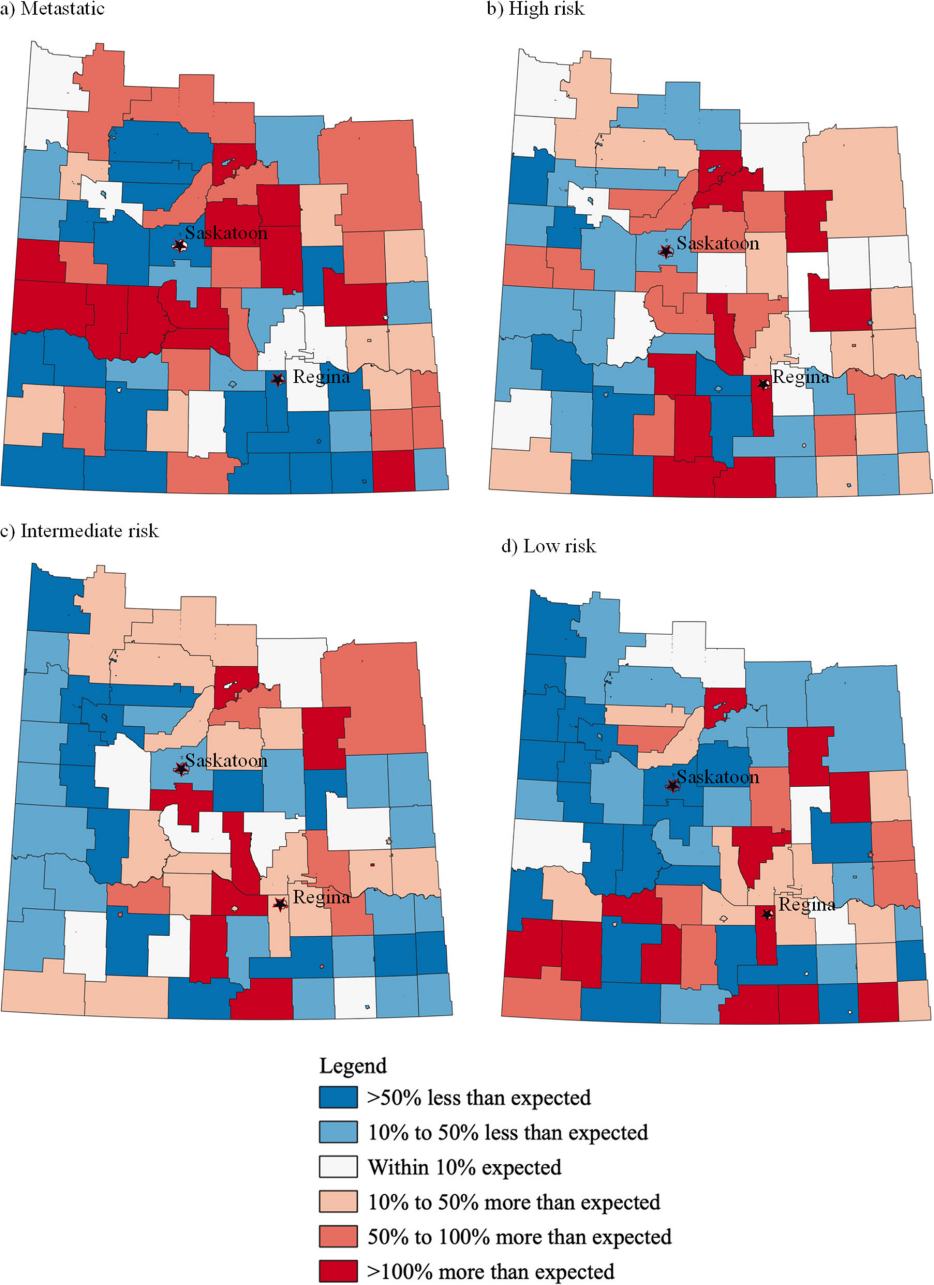
Crude estimated SIRs for metastatic, high-risk, intermediate-risk, and low-risk PCa cases in Saskatchewan (2010–2014)

**Fig. 2:**
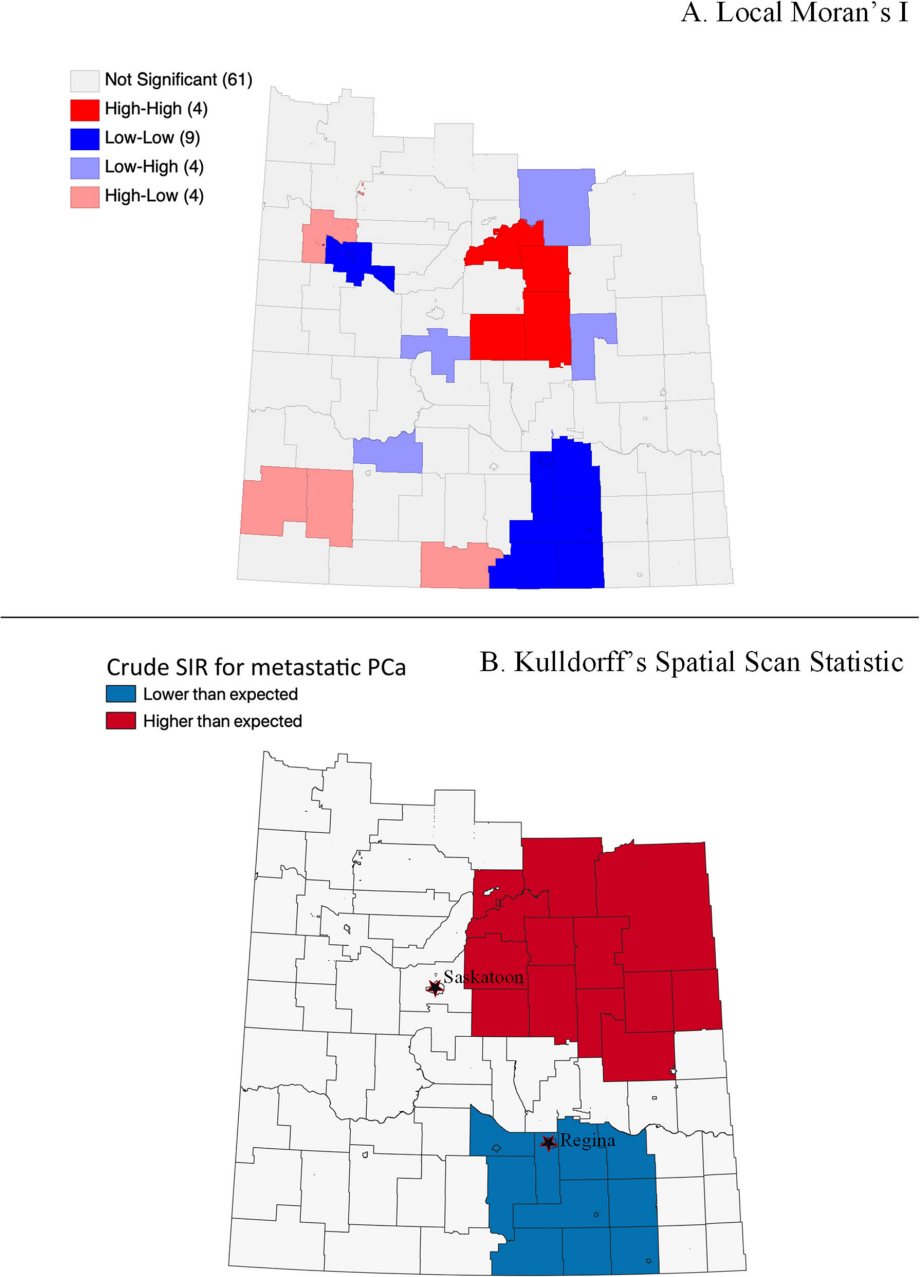
Metastatic PCa crude estimated SIR clustering analysis: (**A**) Local Moran’s I; (**B**) Kuldorff’s Spatial Scan Statistic

The data for PCa were from the Saskatchewan Cancer Registry (SCR) and consisted of demographic, clinical, and geographic information for 3526 patients diagnosed with PCa between 2010 and 2014. Based on the demographic information, all PCa patients were age 35 years or over. The study area contained 82 geographic areas (GAs) in central and southern Saskatchewan categorized (for privacy reasons) by SCR using residence codes (Figs. 1 and 2) [23]. From 2010 to 2014, the study area contained 3289 PCa patients, after excluding those living out-of-province at the time of diagnosis (194 patients) and those (43 patients) living in the three northern regions (Mamawetan Churchill River, Keewatin Yatthe, and Athabasca). The northern regions were excluded because these regions could not be subdivided due to privacy reasons. Of these 3289 PCa patients, the analysis further excluded 298 patients because their PCa risk levels were unknown. Therefore, the final sample had 2991 patients, each categorized per the GA in which the patient lived at the time of diagnosis.

To calculate SIRs (described in the following sections), population counts for 2012 from the Saskatchewan Covered Population (SCP) were used in the denominator in the formula to calculate PCa SIR (see Definitions section) [23]. The SCP is a count of residents with provincial health insurance in Saskatchewan in a given year and is maintained by Government of Saskatchewan [23]. Because all PCa patients in the SCR dataset were age 35 or over, the SCP data used was for men over the age of 35. The overall SCP for men age 35 or over deviated less than 3% each year [24], therefore we chose to use statistics from the midpoint year 2012 (between 2010 and 2014) for the denominators in the calculations [25].

To calculate physician density (described in the following sections) for the period 2010 to 2014, the required data from the Canadian Medical Association was only available for 2011 [26]. Hence our estimated physician densities are based on the year 2011. Canadian Medical Association data consists of family physicians and general practitioners licensed to practice medicine in Saskatchewan.

The relative variability of PCa incidence between the time period (2010 to 2014) was assessed using the coefficient of variation (ratio of the standard deviation to the average).

The University of Saskatchewan BioMedical Research Ethics Board provided ethics approval (Bio-REB certificate #15–34).

### Definitions

The risk levels (low, intermediate, high) for PCa were based on the Genitourinary Radiation Oncologists of Canada (GUROC) definitions [27] and a fourth risk level (“metastatic”) was added to include patients diagnosed with metastatic cancer. For each risk level, the expected number of PCa cases in the i^th^ GA (E_i_) was calculated as follows [28]:

$$ {E}_i={n}_i\left(\frac{\sum_i{O}_i}{\sum_i{n}_i}\right) $$

where n_i_ and O_i_ respectively denote the population count of men age 35 or over and the observed number of PCa cases in the i^th^ GA. For each PCa risk level, the standardized incidence ratio (SIR) (which will be referred to as the crude estimated SIR) was estimated by dividing the number of observed cases in by the number of expected cases in each GA [28].

### Independent variables

The study variables of interest were physician density, remoteness index, and closest PCa assessment centre. For each GA, the family physician density was calculated using the 2011 Canadian Medical Association data and the same population denominator used for the expected count of PCa cases. A remoteness index for a GA was calculated using the average of the Statistics Canada remote indices for regions forming the GA [29]. For each GA, the closest PCa assessment centre was categorized as Regina or Saskatoon, based on the shortest Euclidean distance between the centroid of the GA and the centroids of Saskatoon and Regina. Further details regarding remoteness index and closest PCa assessment centre variables used in this study can be found in the literature [3, 4].

### Statistical methods

Clustering analysis was conducted to identify spatial clusters of PCa SIR by each risk level. Second, for each PCa risk level, a null model was built where the crude estimated SIRs were smoothed using the method proposed by Besag, York and Mollie (BYM model) [29]. The estimated SIRs from the BYM models will be referred to as the smoothed estimated SIRs. Third, ecological analyses were conducted to assess associations between the independent variables and the smoothed estimated SIRs for each of four PCa risk levels.

#### Clustering analysis

For each risk level, Global Moran’s I was calculated using the crude estimated SIR value for each GA [30]. The statistical significance for Global Moran’s I statistic was calculated using 999 permutations [30], which, if significant, demonstrates that the GAs sharing common boundaries have similar SIRs instead of having random geographically-distributed SIRs [31]. GAs within a 120-km radius of a GA were identified as neighbours of the GA. The corresponding weight matrix for the analysis was then computed using the inverse of the Euclidean distances between the centroids of a GA and its neighbours. This weight matrix was chosen to reflect the suspected correlation structure of the data [32].

For each risk level with statistically significant Global Moran’s I values, the crude estimated SIRs were studied further using the Local Moran’s I and Kuldorff’s Spatial Scan statistics [33–35].

#### BYM modeling

The SIRs were estimated using a Bayesian model-based approach to ensure, if spatial correlation exists, the estimated SIRs (i.e the smoothed estimated SIRs) were corrected for any spatial dependence between the GAs.

First, for each PCa risk level, a null model was built where the smoothed estimated SIRs were computed using the Bayesian BYM method [29]. Due to the count nature of the data, we assume our observed data O_i_ follows a Poisson distribution [36] with mean E_i_θ_i_ where E_i_ and θ_i_ respectively denote the expected number of PCa cases and the “true” SIR in the i^th^ GA [37, 38]. The BYM method models the log of the SIR as follows:

$$ \mathrm{Log}\ \left({\uptheta}_{\mathrm{i}}\right)=\mathrm{c}+{\mathrm{u}}_{\mathrm{i}}+{\mathrm{v}}_{\mathrm{i}} $$

where intercept c is the mean, and the terms u_i_ and v_i_ respectively denote the spatially structured and unstructured random effects [37, 38].

The parameters used in this model are based on literature [37–41]. The random effects and the intercept are assigned prior distributions. The intercept was assigned a uniform prior that extends over the whole real line [37, 38]. The structured random effect u_i_ was assumed to follow a conditional auto-regressive distribution and the unstructured random effect v_i_ was assumed to follow a normal distribution with mean zero [37, 38]. The variability for both random effects were controlled by a precision parameter. The precision parameter for the random effects were assigned a Gamma distribution with hyper-prior specification of (0.5, 0.0005) [39, 41].

The simulation for each model consisted of three chains [42, 43]. Each chain consisted of 200,000,000 iterations to obtain 50,000 data points: one for each 4000 time steps taken. A burn-in period of 8,000,000 iterations was selected based on the characteristics of the Brooks-Gelman-Rubin plots [38, 42, 43]. To determine whether the generated estimates for each parameter were from the correct distribution, the following diagnostic tests were performed: potential scale reduction factor, [42] stationarity and half-width tests, [44] Z-score for equality of the means, [45] and run length control [46, 47]).

#### Ecological analysis

Using the BYM models, unconditional analyses were conducted to identify any associations between the independent variables and the SIRs for each risk level. The statistical significance of an independent variable was determined via its 95% credible interval (CrI).

Global and Local Moran’s I statistics were computed using Geoda 1.12 [48]. Kuldorff’s Spatial Scan Statistic was computed using SatScan™ v9.4 [49]. SIR maps were built using quantum Geographical Analysis System (QGIS.org) Version 3.12 [50]. BYM models were built in OpenBUGS version 3.2.3 [51]. Convergence diagnostics for the BYM models were conducted in R using the package ‘coda’ [52].

## Results

The study sample consisted of an average count of 598 PCa cases per year between 2010 and 2014, and the coefficient of variation for PCa incidence between 2010 and 2014 was 7.9%. During the five-year period, the coefficient of variation for remoteness index and physician density were 1.4 and 5.0%, respectively. Based on the age demographic information of all cases, a majority of the PCa cases were 70 years or older, followed by those who were 60 to 69 years old (Table 1). However, the distribution of the age demographics varied by risk level. Low and intermediate risk PCa cases had higher proportions of cases in the younger age groups. In contrast, high risk and metastatic cases had higher proportions of cases in the older age groups. Among all cases, each year (between 2010 and 2014) the proportion of cases diagnosed was about 20% with deviations of less than 2%. See Table 1 for details.

**Table 1:** Demographic information of the PCa cases stratified by GUROC risk levels (*n* = 2991)

	Metastatic	High Risk	Intermediate Risk	Low Risk	Total
**Age**					
Less than 60 years	27 (6.7)	168 (15.4)	277 (26.2)	118 (26.6)	590 (19.7)
v60 to 69 years	87 (21.6)	393 (36.1)	458 (43.4)	224 (50.5)	1162 (38.9)
70 years or older	288 (71.6)	528 (48.5)	321 (30.4)	102 (23.0)	1239 (41.4)
**Year of diagnosis**					
2010	81 (20.2)	193 (17.7)	206 (19.5)	84 (18.9)	564 (18.9)
2011	73 (18.2)	252 (23.1)	211 (19.8)	104 (23.4)	640 (21.4)
2012	58 (14.4)	223 (20.5)	265 (25.1)	113 (25.5)	659 (22.0)
2013	89 (22.1)	213 (19.6)	197 (18.7)	66 (14.9)	565 (18.9)
2014	101 (25.1)	208 (19.1)	177 (16.8)	77 (17.3)	563 (18.8)
**Total**	402 (100.0)	1089 (100.0)	1056 (100.0)	444 (100.0)	2991 (100.0)

The highest proportion of cases were high-risk PCa (36.4%) followed by intermediate-risk (35.3%), low-risk (14.8%) and metastatic cases (13.4%). In nearly a third of GAs (32.9%), the observed incidence of metastatic PCa was more than 50% than the expected incidence. In 28, 18 and 24% of GAs, the observed incidences of high-risk, intermediate-risk and low-risk PCa, respectively, were more than 50% than the expected incidence. See Table 2 for details.

**Table 2:** Crude estimated SIRs for PCa cases diagnosed within geographic areas by GUROC risk level

	GUROC Risk Level			
	Metastatic	High Risk	Intermediate Risk	Low Risk
**Crude estimated SIR by Geographic Areas**				
> 50% less than expected	21 areas	9 areas	16 areas	24 areas
10 to 50% less than expected	15 areas	25 areas	23 areas	17 areas
Within 10% expected	10 areas	12 areas	9 areas	6 areas
10 to 50% more than expected	9 areas	13 areas	19 areas	15 areas
50 to 100% more than expected	15 areas	13 areas	7 areas	8 areas
> 100% more than expected	12 areas	10 areas	8 areas	12 areas

### Clustering analysis

The pattern of crude estimated SIRs for each PCa risk level in Saskatchewan is visualized in Fig. 1. Spatial patterns within Fig. 1 are identified using clustering analysis. The Global Moran’s I statistics for the crude estimated SIRs for each PCa risk level (except for high-risk) show evidence of positive spatial autocorrelation (Table 3). Hence, there was evidence that some geographical areas in Saskatchewan sharing boundaries have similar crude estimated SIRs for metastatic, intermediate and low risk PCa, instead of a random distribution of incidence patterns.

**Table 3:** Result of the Global and Local Moran’s I for each of GUROC risk level

	Metastatic	High Risk	Intermediate Risk	Low Risk
**Global Moran’s I statistic**	0.132*	0.058	0.128*	0.106*
**Local Moran’s I**				
High-High	4 areas	–	8 areas	2 areas
Low-Low	9 areas	–	13 areas	15 areas
Low-High	4 areas	–	0 areas	4 areas
High-Low	4 areas	–	2 areas	1 area
Not Significant	61 areas	–	59 areas	60 areas

*statistically significant at 5% level of significance

**Fig. 3:**
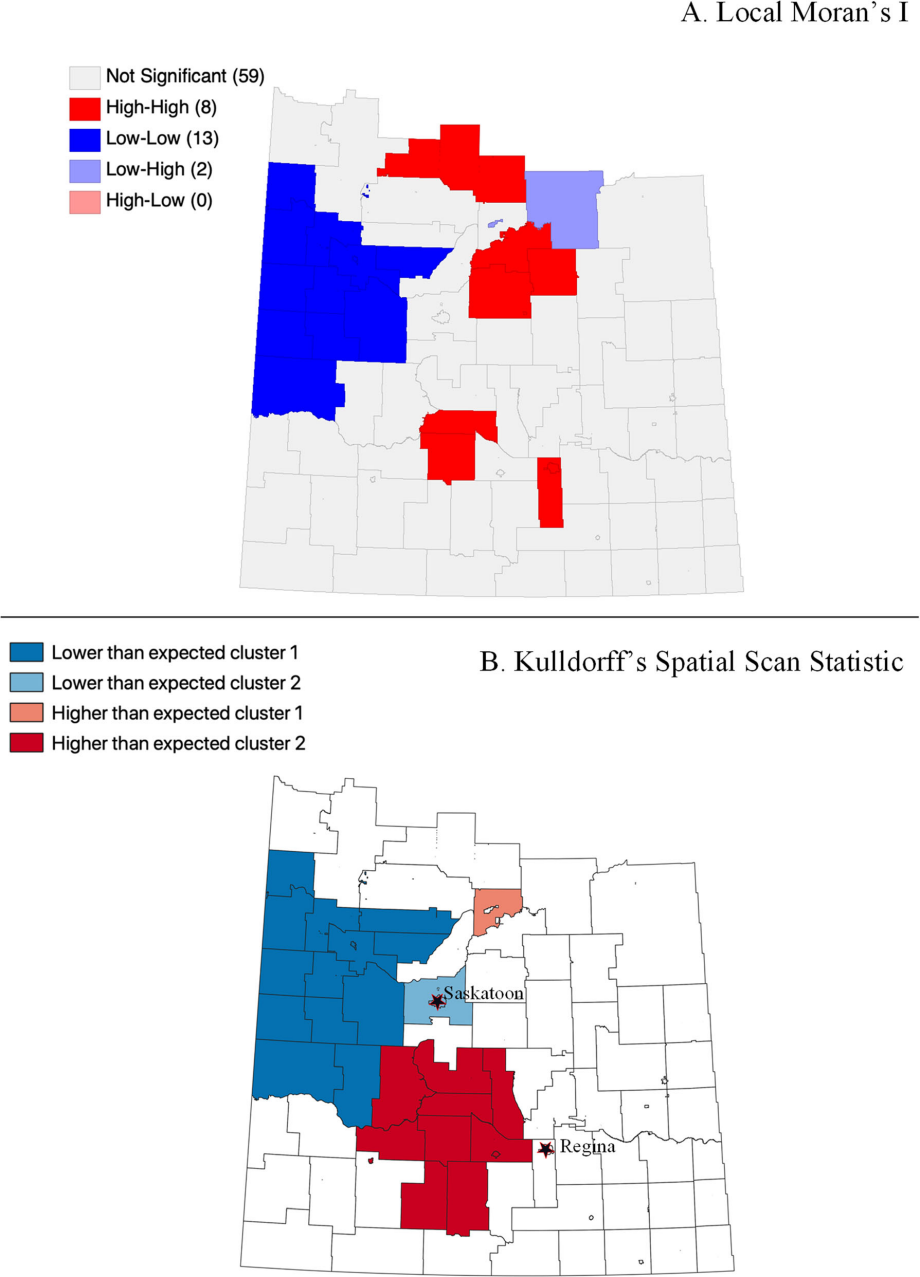
Intermediate-risk PCa crude estimated SIR clustering analysis: (A) Local Moran’s I; (**B**) Spatial Scan Statistic

**Fig. 4:**
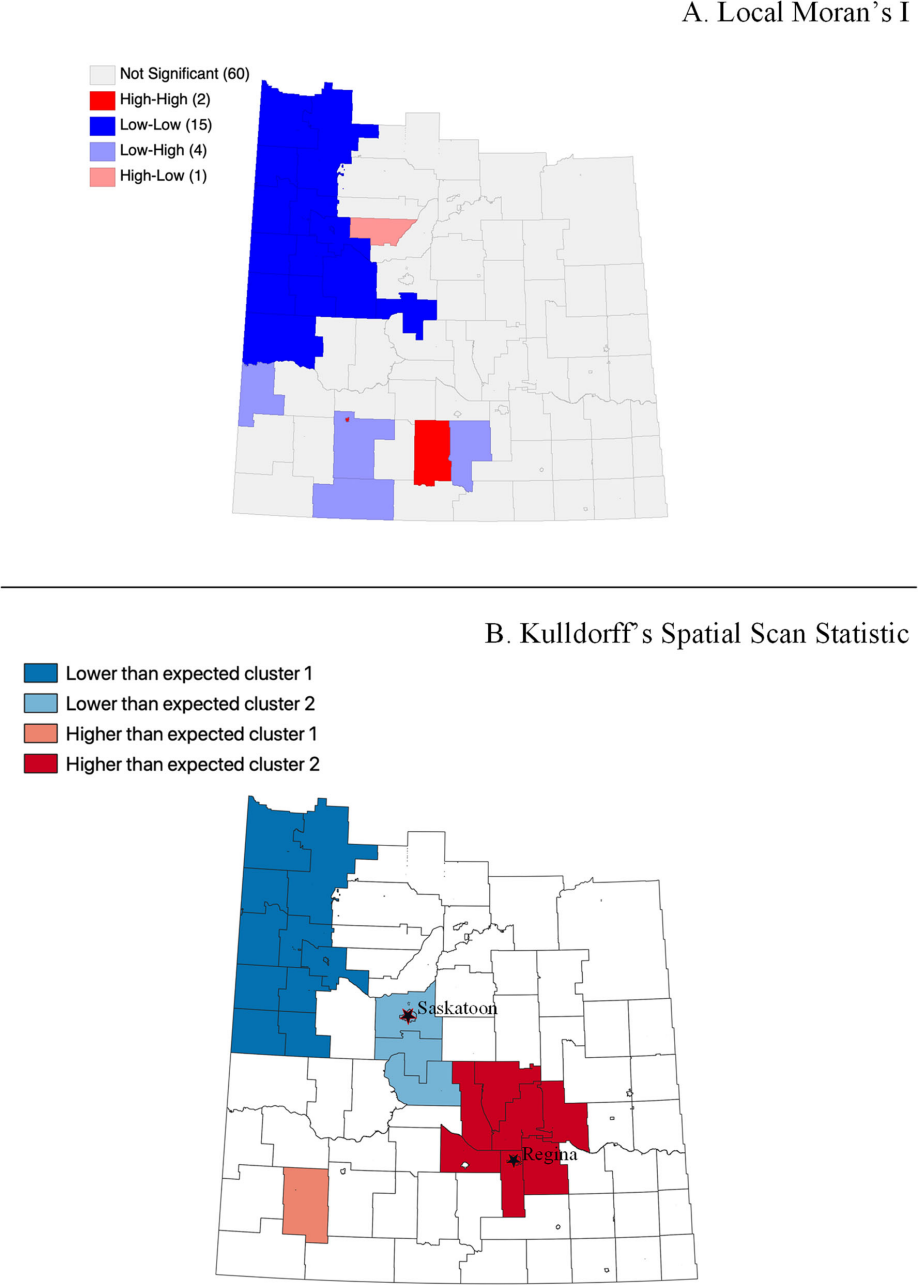
Low-risk PCa crude estimated SIR clustering analysis: (**A**) Local Moran’s I; (**B**) Spatial Scan Statistic

Using the Local Moran’s I statistic, clusters of crude estimated SIRs for each PCa risk level were identified. In Fig. 2, “high-high” clusters of metastatic PCa were identified in the north-east part of the study area. Hence areas in north-east Saskatchewan have higher-than-average crude estimated SIRs for metastatic PCa. For intermediate-risk and low-risk PCa, “low-low” clusters are identified in the north-west part of the study area. Therefore, areas in north-west Saskatchewan have lower-than-average crude estimated SIRs for both intermediate-risk and low-risk PCa (Fig. 3 and Fig. 4).

For Kuldorff’s Spatial Scan Statistic, the maximum spatial window size for metastatic, intermediate and low risk PCa were equal to or less than 30, 25 and 25%, respectively, of the total population. Kuldorff’s Spatial Scan Statistic identified a higher-than-the-average cluster of crude estimated SIRs for metastatic PCa in north-east Saskatchewan and lower-than-the-average cluster in south-east Saskatchewan, analogous to the clusters identified using the Local Moran’s I statistics (Fig. 2). Similarly, the spatial scan statistic results for intermediate-risk and low-risk PCa were comparable to the clusters identified using the Local Moran’s I statistics described earlier (Fig. 3 and Fig. 4).

### BYM modeling

**Fig. 5:**
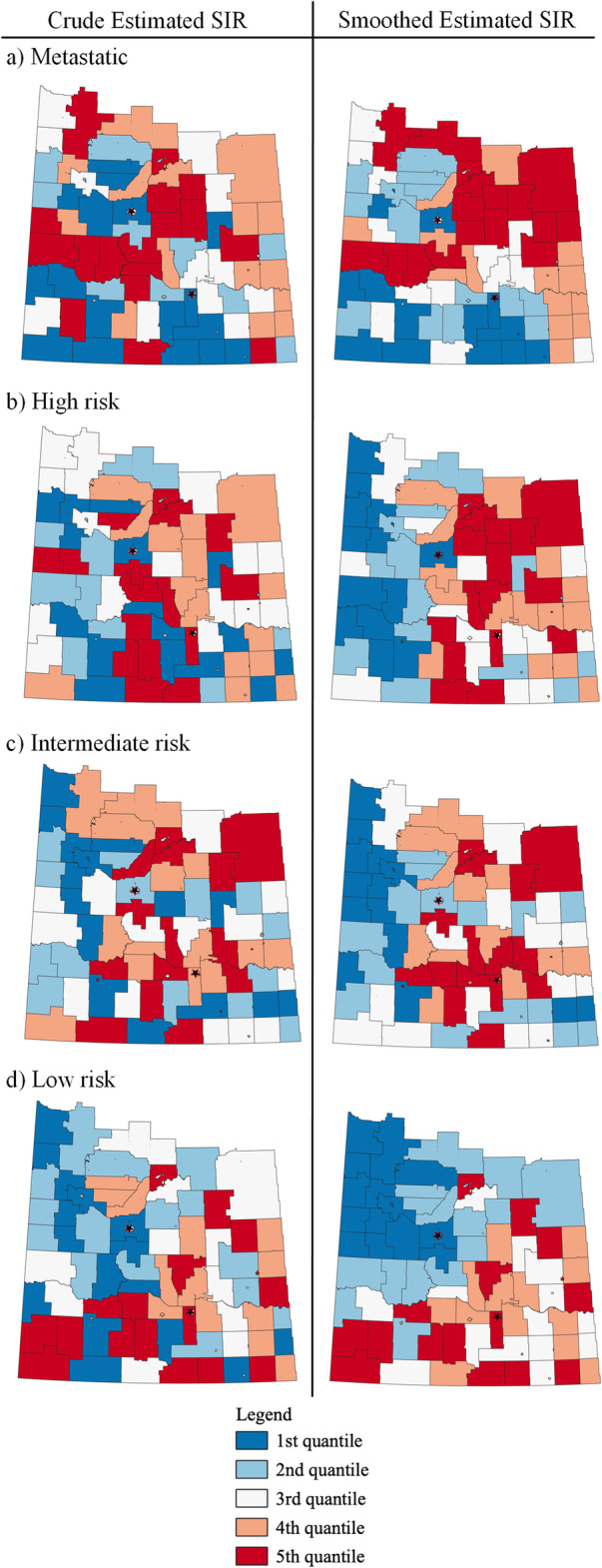
Quantile distribution of PCa crude estimated SIR and smoothed estimated SIR by GUROC risk levels

The crude and smoothed estimated SIRs for a GA are illustrated in Fig. 5. For both metastatic and high-risk PCa, the smoothed BYM estimates highlight areas of elevated incidence in north-east part of Saskatchewan (Fig. 5). Also in Fig. 5, the smoothed estimated SIRs for intermediate-risk and low-risk PCa identify areas of low incidence in north-west part of Saskatchewan. Table 4 illustrates how the crude estimated minimum and maximum SIR values are adjusted by the BYM modelling.

**Table 4:** Comparison of minimum/maximum values of crude and smooth estimated SIRs for each GUROC risk level

	Crude estimated SIRs		Smoothed estimated SIRs	
Outcome	Min	Max	Min	Max
Metastatic	0.000	3.474	0.921	1.221
High Risk	0.213	3.633	0.883	1.218
Intermediate Risk	0.000	5.888	0.347	3.093
Low Risk	0.000	4.470	0.235	2.348

### Ecological analysis

**Fig. 6:**
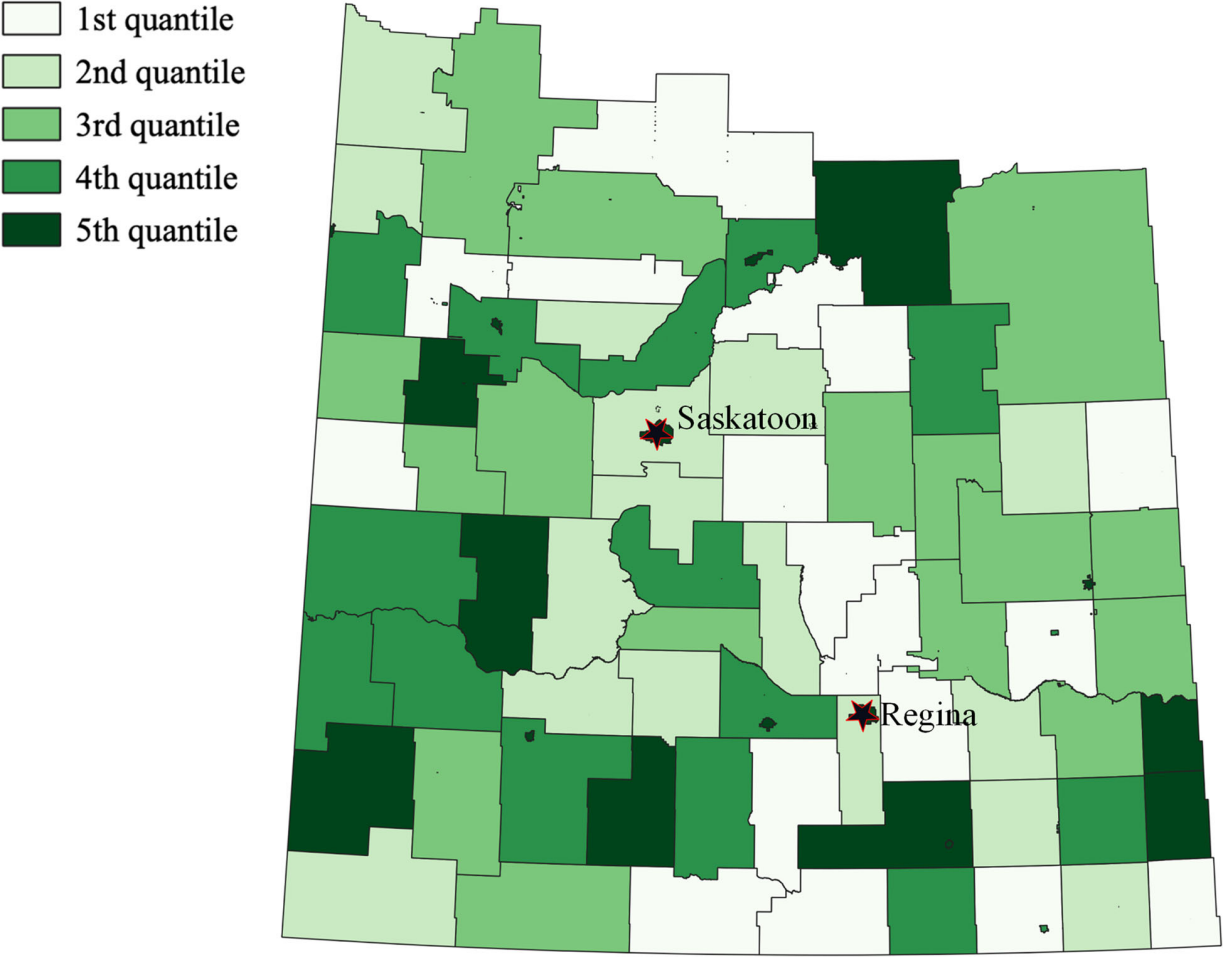
Quantile distribution of family physician density in Saskatchewan in 2011

Family physician density was negatively associated with the smoothed estimated SIRs for metastatic PCa (IRR: 0.935 [CrI: 0.880 to 0.998]) and for high-risk PCa (IRR: 0.927 [CrI: 0.880 to 0.975]). Based on the mean coefficient of family physician density for metastatic PCa (Table 5), one unit increase (or increase of 1 physician per 1000 population) would be equal to an average decrease in metastatic PCa SIR by 6.5%. Similarly, an average increase of 1 physician per 1000 population would be equal to an average decrease in high-risk SIR by 7.3%. Figure 6 provides geographic pattern of family physician density in Saskatchewan and comparison with Fig. 5 visually compliments the negative correlation with metastatic PCa and high-risk PCa. For intermediate-risk and low-risk PCa, based on the credible intervals, there was no evidence of association with family physician density (Table 5).

**Table 5:** Result of the ecological analysis using Bayesian BYM analysis for each of GUROC risk level

	Physician Density (Number of physicians per 1000 population)		
Outcome	Mean	Credible Interval	Incidence Rate Ratio (Credible Interval)
Metastatic	−0.067	− 0.128 to − 0.002	0.935 (0.880 to 0.998)
High Risk	− 0.076	− 0.128 to − 0.025	0.927 (0.880 to 0.975)
Intermediate Risk	− 0.041	− 0.109 to 0.026	Not significant
Low Risk	− 0.009	− 0.079 to 0.062	Not significant

There was no evidence of any association between the SIR of each PCa risk levels and the two remaining independent variables (closest PCa assessment centre and remoteness index).

## Discussion

This study estimated risk stratified PCa SIRs in Saskatchewan to identify if any geographic patterns and disparities. The geographic patterns of the risk stratified SIRs identified areas of concern (higher than expected SIRs) in Saskatchewan using Bayesian models and traditional clustering analysis methods. This study found clustering of higher than expected incidence for metastatic PCa in north-east part of Saskatchewan, and lower than expected incidence in south-east part of Saskatchewan. This study also identified lower than expected incidence of intermediate-risk and low-risk PCa in the north-west part of Saskatchewan. The estimation of SIRs using the BYM method led to adjustment of the crude estimated SIRs to facilitate identification of spatial trends [53].

Our study also shows that areas with lower density of family physician have higher than expected incidence of metastatic and high-risk PCa. A similar trend has been observed in the United States where increases in primary care physician density were associated with a decrease in late-stage diagnosis of cancers including PCa [21, 54]. The findings of this study highlight the effect that increasing physician supply may have on improving health outcomes, as identified in previous studies including PCa [14–20]. The results also highlight the wide-ranging distribution of family physician within Saskatchewan, acknowledging Saskatchewan also has one of the lowest per capita physician supply compared to the other provinces in Canada [13]. Hence, policies to increase physician supply should ensure equitable geographic distribution of primary care physicians to support early detection of diseases including PCa.

Nearly half of the patients in the sample were high risk (36.4%) or metastatic (13.4%), which could be indicative of physician practices including receptiveness towards PCa screening policies. Literature shows that physician beliefs regarding PCa screening/diagnosis procedures can influence their practice (physicians who are uncertain about PCa screening/diagnosis procedures are less receptive towards offering PCa screening/diagnosis to their patients). Because such practice variations exist among Saskatchewan physicians [55] and the literature reports that an increase in advanced PCa may be due to a decrease in PCa screening [56], physician beliefs might possibly explain the geographic variations in PCa diagnosis rates.

Although family physician density was not associated with diagnostic pattern for low-risk and intermediate-risk PCa, further research is needed if these regional trends are related to physician practices given the controversy of screening tests for early detection of PCa [57, 58]. Given recent research showing PCa screening and detection of early-stage PCa decreasing, potentially due to mixed PCa screening guidelines, further studies assessing the role of PCa screening guidelines on geographic disparities in early-stage PCa incidence may provide further explanation [59].

This study identifies the geographical disparities in risk-stratified PCa incidence in Saskatchewan. This study suggests that healthcare access factors [60], including availability of physicians and the geographic location of individuals, may affect health outcomes for PCa. This study further highlights the possibility that enhancing health delivery in rural areas may improve health outcomes. A recent report by the Rural Road Map Implementation Committee in Canada shows there are continued challenges regarding healthcare access in rural parts of Canada including difficulties of attracting and retaining physicians [61].

The limitations of this study include the use of aggregate data for the ecological study design due to lack of information on individual-level data on family physician availability to the patient. However, the study uses widely developed Bayesian and conventional spatial analysis methods to identify inherent patterns in the study area. Other limitations include the unavailability of socioeconomic/demographic data and PCa screening data for the study period.

## Conclusions

This study identified geographic disparities in PCa incidence in Saskatchewan. There were higher than expected incidence of metastatic PCa in north-east parts of Saskatchewan, and lower than expected incidence of intermediate-risk and low-risk PCa in the north-west part of Saskatchewan. In addition, areas with lower density of family physician had higher than expected incidence of metastatic and high-risk PCa. This study shows that availability of community level healthcare providers and geographic location of patients affects cancer care in Saskatchewan. This highlights the need for adequate availability of primary care physicians in rural and urban areas to improve cancer care in Saskatchewan.

## Data Availability

This study was conducted secondary analysis of existing data, which is only available at Saskatchewan Cancer Registry. For further information on accessing data, please contact Saskatchewan Cancer Registry at info@saskcancer.ca.

## Abbreviations

PCa: prostate cancer
SIRs: standardized incidence ratios
BYM: Besag, York and Mollie
GA: Geographic Areas
IRR: Incidence Rate Ratio
CrI: Credible Interval
SCR: Saskatchewan Cancer Registry
SCP: Saskatchewan Covered Population
GUROC: Genitourinary Radiation Oncologists of Canada
